# Supplementary material for: Analysis of the mutant selection window and killing of Mycoplasma hyopneumoniae for doxycycline, tylosin, danofloxacin, tiamulin, and valnemulin
Source: PLoS One. 2020 Jun 16;15(6):e0220350. doi: 10.1371/journal.pone.0220350 (PMC7297357; doi:10.1371/journal.pone.0220350)
Supplement: Supplementary file 1 [file pone.0220350.s001.docx]

Time-killing of 10^5 CFU/mL

Time	Drug free	Dnaofloxacin MIC_{99}	Tiamulin MIC_{99}	Tylosin MIC_{99}	Doxcycline MIC_{99}	Valnemulin MIC_{99}
0	450000	430000	480000	560000	520000	410000
3	622000	267000	411000	633000	238000	385000
6	522000	142000	272000	433000	173000	159000
9	778000	44400	183000	311000	156000	146000
12	656000	15600	233000	88900	102000	40000
24	1470000	178	144000	7330	90000	4440
36	1640000	100	4330	100	13400	100
48	2330000	100	100	100	21000	100
Time	Drug free	Dnaofloxacin MPC	Tiamulin MPC	Tylosin MPC	Doxcycline MPC	Valnemulin MPC
0	450000	430000	480000	560000	520000	410000
3	622000	29300	170000	145000	113000	90000
6	522000	3330	83300	117000	100000	83300
9	778000	3170	63000	133000	41700	43300
12	656000	1500	35000	43300	22000	3330
24	1470000	100	2670	1250	783	150
36	1640000	100	100	100	200	100
48	2330000	100	100	100	100	100

			Time-killing of 10^7 CFU/mL			
Time	Drug free	Dnaofloxacin MIC_{99}	Tiamulin MIC_{99}	Tylosin MIC_{99}	Doxcycline MIC_{99}	Valnemulin MIC_{99}
0	13300000	14100000	16300000	13600000	14900000	12300000
3	20000000	2870000	22300000	17200000	4170000	3670000
6	15000000	2330000	28900000	12500000	3780000	2890000
9	26700000	378000	4780000	12300000	1670000	1770000
12	24400000	111000	3830000	2890000	900000	722000
24	26700000	2570	1570000	3720000	622000	65600
36	46700000	344	63300	291000	408000	533
48	78900000	100	5000	1000	1020000	100
Time	Drug free	Dnaofloxacin MPC	Tiamulin MPC	Tylosin MPC	Doxcycline MPC	Valnemulin MPC
0	13300000	14100000	16300000	13600000	14900000	12300000
3	20000000	517000	1500000	2300000	1170000	1970000
6	15000000	300000	1770000	1080000	172000	700000
9	26700000	200000	800000	600000	68300	250000
12	24400000	71700	967000	250000	50000	143000
24	26700000	11700	11200	46000	40000	12500
36	46700000	100	2000	1000	46700	1500
48	78900000	100	100	100	1020	100

			Time-killing of 10^9 CFU/mL			
Time	Drug free	Dnaofloxacin MIC_{99}	Tiamulin MIC_{99}	Tylosin MIC_{99}	Doxcycline MIC_{99}	Valnemulin MIC_{99}
0	1030000000	1080000000	1130000000	1150000000	1010000000	1170000000
3	1022000000	43400000	137800000	74600000	36000000	38400000
6	1866000000	15120000	97800000	45800000	32000000	22200000
9	1556000000	7780000	51600000	68200000	21000000	28000000
12	2300000000	4660000	75600000	43600000	19500000	10800000
24	1778000000	320000	57400000	37600000	14300000	5330000
36	2340000000	44400	54600000	4460000	4000000	467000
48	1054000000	2660	6660000	4660000	3750000	18300
Time	Drug free	Dnaofloxacin MPC	Tiamulin MPC	Tylosin MPC	Doxcycline MPC	Valnemulin MPC
0	1030000000	1080000000	1130000000	1150000000	1010000000	1170000000
3	1022000000	10000000	40000000	20000000	53750000	21200000
6	1866000000	1230000	26600000	7000000	12300000	16200000
9	1556000000	400000	24000000	5630000	5400000	1680000
12	2300000000	88300	4750000	5000000	4400000	517000
24	1778000000	25000	375000	4250000	2400000	400000
36	2340000000	1200	640000	145000	120000	73300
48	1054000000	100	83300	50000	110000	14700

**Liquid method**			
	10^5 CFU/mL	10^6 CFU/mL	10^7 CFU/mL
Danofloxacin	0.03125	0.0625	0.125
Timulin	0.04	0.08	0.08
Tylosin	0.05	0.1	0.2
Doxycycline	0.125	0.125	0.25
Valnemulin	0.002	0.002	0.004

**Solid method**			
	10^5 CFU/mL	10^6 CFU/mL	10^7 CFU/mL
Danofloxacin	0.125	0.125	0.25
Timulin	0.16	0.160	0.32
Tylosin	0.40	0.40	0.80
Doxycycline	0.50	0.500	1.00
Valnemulin	0.008	0.016	0.016
